# Risk Factors for Workplace Bullying: A Systematic Review

**DOI:** 10.3390/ijerph16111945

**Published:** 2019-05-31

**Authors:** Fernando R. Feijó, Débora D. Gräf, Neil Pearce, Anaclaudia G. Fassa

**Affiliations:** 1 Postgraduate Programme in Epidemiology, Department of Social Medicine, Federal University of Pelotas, 96030-000, Brazil; dalmasgraf@gmail.com (D.D.G.); anaclaudia.fassa@gmail.com (A.G.F.); 2 Centre for Health Sciences, Federal University of Recôncavo da Bahia, 44574-490, Brazil; 3 Department of Medical Statistics, Faculty of Epidemiology and Population Health, London School of Hygiene and Tropical Medicine, London WC1E 7HT, UK; neil.pearce@lshtm.ac.uk

**Keywords:** workplace bullying, occupational health, epidemiology

## Abstract

*Objective*: The goal of this study was to systematically review risk factors for workplace bullying. *Methods*: The search was carried out in two databases. Studies with estimates of risk factors for workplace bullying were included in the review. We assessed the quality of the selected studies using an adapted version of the Downs and Black checklist. Preferred Reporting Items for Systematic Reviews and Meta-Analyses (PRISMA) and Meta-analyses of Observational Studies in Epidemiology (MOOSE) guidelines were used for reporting papers. *Results*: Fifty-one papers were included, and 70.6% were from European countries. Women were reported to be at higher risk of being bullied in most studies (odds ratio (OR) from 1.17 to 2.77). Authoritarian and laissez-faire leadership styles were positively associated with bullying. Several psychosocial factors, such as stress (OR from 1.37 to 4.96), and occupational risks related to work organization, such as flexible work methods, role conflict, role ambiguity, monotonous or rotating tasks, high demands, pressure of work, and unclarity of duties were strongly associated with bullying. *Discussion*: The findings highlight the central role of organizational factors in bullying. Policies to prevent bullying must address the culture of organizations, facing the challenge of developing a new management and leadership framework.

## 1. Introduction

Workplace bullying is a still relatively recent issue in occupational health research, with most studies having occurred in the last 30 years. It is defined as situations where a person repeatedly and over a period of time is exposed to harassment, abuse, offenses, or social exclusion, placing the individual in an asymmetrical position where he/she is not able to defend himself/herself from unethical behaviour [1,2,3,4]. Its occurrence in workplaces is high, as demonstrated by a systematic review of 102 estimates, which identified an overall prevalence of 14.6% [1]. Also, bullying can be considered one of the greatest threats for workers’ well-being [5,6], reinforcing the importance of better understanding its causes and mechanisms.

In the 1990s, Leymann claimed that four main factors were related to the occurrence of bullying in organizations: deficiencies in work design, deficiencies in leadership behaviour, socially exposed position of the victim, and low moral standards in the organization [2,3,4], describing the ‘work environment hypothesis’. Since then, several studies have been conducted in order to elucidate its impact and causes. Recent systematic reviews and longitudinal studies of the consequences of workplace bullying demonstrate that bullying is associated with mental health problems—such as depression [7], anxiety [8,9], suicidal ideation [10,11]—sleep problems [12,13], neck and back pain [14], cardiovascular disease [15], diabetes [16], and absenteeism [17]. However, as a multicausal and complex phenomenon [4], effectively reducing bullying in workplaces is still a challenge. A recent Cochrane review established that there is very low-quality evidence regarding interventions to prevent bullying at work [18].

Since the 1990s, a few studies have attempted to summarize the risk factors for workplace bullying. Two reviews published in 2006 assessed antecedents of workplace bullying in the general working population [19,20]. These reviews included very few studies [19] or focused on specific risk factors such as role conflict and role ambiguity [20], and only reported correlation coefficients. Since then, many new articles regarding risk factors for bullying have been published. Another two recent reviews assessed exclusively the relationship between personality traits and bullying [21], and reviewed risk factors for bullying in nurses [22]. Nevertheless, no reviews have focused on epidemiological studies or estimates of risk factors for bullying.

Therefore, there is still a need to identify the most important risk factors for workplace bullying to estimate their effects, to develop occupational health policies, and to guide theoretical models and research hypotheses on this subject. Thus, the aim of this study was to systematically review epidemiological findings on risk factors for workplace bullying and contribute to its theoretical framework.

## 2. Materials and Methods

We carried out an electronic search on the Medline (PubMed) and the Latin American Centre for Health Sciences Information (BIREME) (which includes the *Literatura Latino-Americana e do Caribe em Ciências da Saúde* (LILACS), *Índice Bibliográfico Espanhol de Ciências de Saúde* (IBECS), *Bibliografía Nacional em Ciencias de La Salud* (BINACIS), *Base de Dados Enfermagem* (BDENF), *Index Psicologia*, *Publicações da Organização Mundial da Saúde* (WHOLIS), *Literatura em Ciências da Saúde dos países do Caribe* (MedCarib) and *Coleciona* SUS) databases. The search was done in September 2018. The review focused on workplace bullying studies using the following keywords: *((((“bullying”[MeSH Terms] OR “bullying”[All Fields]) OR mobbing[All Fields]) OR harassment[All Fields]) OR “negative acts”[All Fields]) AND (((“workplace”[MeSH Terms] OR “workplace”[All Fields]) OR (“work”[MeSH Terms] OR “work”[All Fields])) OR job[All Fields]).*

The identified articles investigated definitions, models, risk factors, and outcomes for workplace bullying. The inclusion criteria included descriptive and analytical studies that evaluated risk factors for workplace bullying from any country worldwide, published in English, Spanish, or Portuguese, from January 1958 to September 2018.

The search and selection of papers were carried out by two researchers. Both researchers were PhD students in a public university and were supported by a governmental scholarship. The first researcher had already worked in the private and public sector, as well as a consultant for private companies and trade unions, and the second researcher did not have any previous occupational experiences. The quality of papers was blindly assessed using an adaptation of the Downs and Black [23] checklist for observational studies, building an index based on 15 items (from 27 items in the original tool). Evaluated points included reporting (5 items), external validity (3 items), bias (5 items), confounding (1 item), and power (1 item). Answers were scored 0 or 1 and the maximum score was 15. We excluded 12 items from the original version as they were focused on clinical trials. For the same reason, the score of item 27 was also modified to zero or one.

The Preferred Reporting Items for Systematic Reviews and Meta-Analyses (PRISMA) [24] and the Meta-analyses of Observational Studies in Epidemiology (MOOSE) [25] were used as guidelines for reporting the selected articles.

## 3. Results

**Figure 1:**
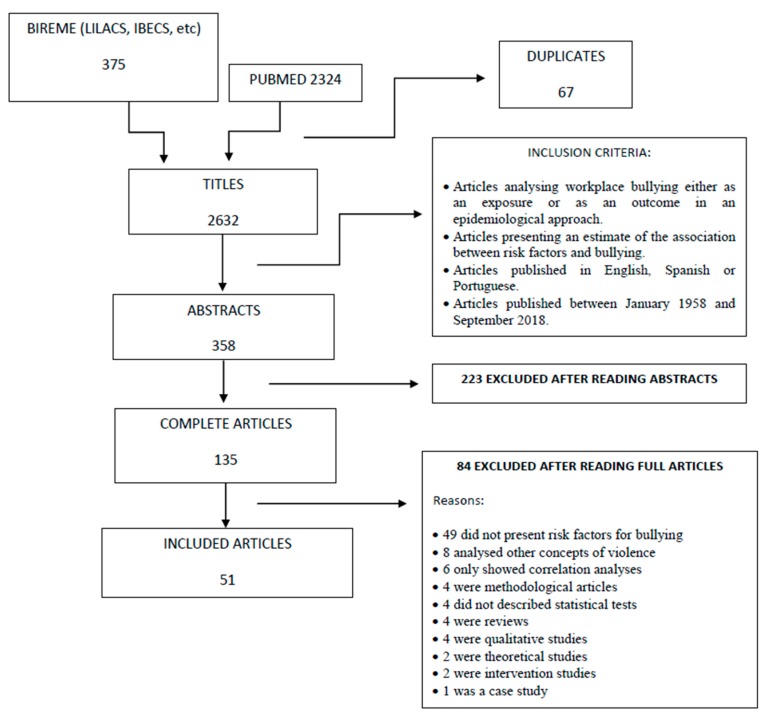
Preferred Reporting Items for Systematic Reviews and Meta-Analyses (PRISMA) flow diagram: studies selection about risk factors for workplace bullying.

The search identified, after exclusion of duplicates, a total of 2632 articles. After reading the titles and abstracts, 135 articles were selected for full reading (Figure 1). A total of fifty-one papers were included in this review (Table 1). The main reasons for exclusion were not presenting risk factors for bullying, analysing other concepts of violence, not describing statistical tests or showing only correlation coefficients, and being methodological articles or reviews. A total of 70.6% (n = 36) of the included studies were from Europe, 11.8% (n = 6) from North America, 9.8% (n = 5) from Australia and New Zealand, and 7.8% from other countries—Japan (n = 2), Mexico (n = 1), and Ghana (n = 1). Regarding methodological issues, most of the studies were cross-sectional, with data either from national surveys or specific groups of workers. Response rates varied from 12.5% to 95.0%, but only two studies reached a response rate higher than 80%. The approaches, methods, objectives, and quality of these studies were very heterogeneous. Papers scored between 6 and 15 points in the Downs and Black assessment tool [23]. A total of 15 articles (29.4%) scored less than 10 points and 18 (35.3%) scored above 13. Missed points in the Downs and Black index were mostly due to lack of losses description, *p*-value reporting, and confounding evaluation.

Most of the studies reported that women are more likely to be bullied than men [26,27,28,29,30,31,32,33,34,35,36,37,38]. Statistically significant odds ratios and prevalence ratios ranged from 1.17 to 2.77. Also, 11 studies showed no association between gender and bullying [39,40,41,42,43,44,45,46,47,48,49]. Only two studies suggested a higher risk among men [50,51]. The results on the association between age and workplace bullying were also inconsistent. Although eight studies found that workers younger than 44 years of age are more likely to be bullied [30,32,37,41,45,50,52,53], nine studies showed no association between age and workplace bullying [29,36,39,42,43,44,47,48,49]. Only one study suggested a higher prevalence of bullying in older workers [54]. 

Only five studies presented relevant data on the association between ethnicity and bullying. Three studies suggested a higher prevalence of bullying between ‘non-white’, ‘multiracial individuals’, and ‘Asian and black’, compared to white workers. The risk was increased in a range from 1.30 to 2.30 times [29,34,50]. One study showed no differences in the Negative Acts Questionnaire (NAQ) mean score when comparing white workers and black or ethnic minorities [51], while one study showed 28% less prevalence of bullying in non-white workers [52]. The NAQ is a tool with several questions about negative acts that suggest workplace bullying during the previous six months [4].

The association between family structure and bullying also varied between studies. Four studies reported no association between workplace bullying and marital status [37,43,49,55]. On the other hand, three studies reported that single, separated, divorced, and widowed workers were more likely to suffer from bullying at work [34,46,56], while one study found a higher risk of bullying among married workers (odds ratio (OR) = 3.06 (1.41–12.94)) [42]. Two studies also found a strong risk of being bullied among workers who have small children at home (OR from 1.92 to 2.87) [35,36].

Most of the studies reported no association between education and bullying. However, data from the 5th European Working Conditions Survey suggested a strong association between lower educational level and bullying (OR = 5.51 (1.79–16.95)) [36]. At the same time, two Turkish studies with forest engineers reported a higher prevalence of bullying among those with a doctoral degree [37,55].

The relationship between personality traits and being a victim of bullying was also evaluated by a few studies. Five studies described statistically significant associations between some specific personality traits and bullying. Neuroticism was identified as a risk factor for bullying in three studies, with odds ratios from 1.23 to 1.28 [26,40,76]. Most of the personality traits evaluated by these studies were not associated with bullying. In a cluster of participants of one study, people with Type A personality [48], less extroverted, less agreeable, less conscientious, less open to experience and more emotionally unstable were also more likely to be bullied [76]. Nevertheless, another study showed that personality characteristics explained only 2% of the variance (adjusted R^2^ = 0.02) of bullying [58].

A wide range of important occupational factors regarding work organization, management issues, type of job, and earnings were also evaluated across the studies. The occurrence of bullying varied across professions, and it was not possible to identify a pattern in this aspect. The results were also controversial concerning the type of work (if permanent or temporary). Civil servants were more likely to be bullied than other workers [34,38,45]. Workers with lower income [63], paid hourly [34], and less satisfied with their payment [35] were also more likely to be bullied. Regarding the association between years of work in the organization and the occurrence of bullying, studies showed either a positive association [43] or no difference [49]. Shift work was strongly and positively associated with workplace bullying. Odds ratios (OR) of this association ranged from 1.74 (US workers) [34] to 2.68 (Spanish workers) [36]. The magnitude of the association was stronger in the public (OR = 2.46) than in the private sector (OR = 1.94) [35]. 

Organizational change, lack of procedural justice [53], and poor psychosocial safety climate were strongly and positively associated with bullying [71]. Leadership style was reported as an important risk factor by nine studies. Passive laissez-faire leadership, evaluated by three articles, increased up to 4.3 times the risk of workplace bullying [64,66,69]. Destructive [61], dictatorial [53], and autocratic leadership [74] were also related to a higher occurrence of bullying. On the other hand, supportive leadership style [57], consideration of individual by leaders [64], transformational and transactional leadership [66], authentic leadership [69], and fair leadership [73] reduced up to 70% the risk of bullying.

Flexible work methods [33], role conflict, role ambiguity [68,72,75], personal conflicts [70,72], less satisfaction with working conditions [35,36], either monotonous or rotating tasks [36], high demands at work, pressure of work, and unclarity of duties [74] were also positively associated with workplace bullying.

Work stress was one of the most important occupational factors reported in empirical studies, and was always strongly and positively related to bullying [33,35,36,49,62]. The odds ratios varied from 1.38 [49] to 4.96 [36]. Lack of social support [30,53,70,72], low social capital [59], and effort–reward imbalance [60] were also strongly associated with a higher risk of bullying.

Lastly, worse physical and mental health increased the risk of being bullied both in cross-sectional and longitudinal analyses. Sick leave and being on sickness treatment were also positively associated with bullying [38,57,61,65,67].

## 4. Discussion

A total of 49 out of 51 studies on risk factors for workplace bullying came from high-income countries, particularly Europe. Thus, either epidemiological studies on workplace bullying have not been developed in low- and middle-income countries, or their findings have not been published internationally. In a globalized scenario, it is not plausible that this phenomenon is not happening in other countries, mainly in countries where working conditions are poor. Only two studies [42,74] had response rates higher than 80%. As Nielsen (2010) advised, representative and convenience samples provide significantly different estimates of the prevalence of bullying [1], which could distort effect measures. On the other hand, most of the studies measured bullying with validated instruments, which tend to provide a ‘more objective’ measure. Although a few adaptations of Leymann’s inventory were described, the NAQ (77) has been used and validated in several working populations [77,78], thus it is an option that improves the comparability of results. Some studies also measured bullying ‘subjectively’ (self-labelled approach), providing its definition and asking whether the worker was bullied or not in a single question. A combination of both objective and subjective measures can also be a satisfactory method to improve specificity [74,79].

The identified studies evaluated workers in several professions and activities, with a great variability of sociodemographic profiles and occupational characteristics, which makes it difficult to summarize and compare the results. While some studies focused on sociodemographic determinants and personality traits as antecedents of bullying, others focused on managerial and organizational factors. The ‘work environment hypothesis’ proposed by Leymann in the early 1990s targeted the work organization as the main cause of bullying, while more recent studies tested new hypotheses regarding the relationship between individual factors, such as personality and bullying [21]. This demonstrates the complexity of the phenomenon and indicates that simple explanations focusing on one or a few aspects of bullying are not enough to study this theme [80].

Regarding demographic factors, the role played by women in a globalized labour market, the work organization of institutions, and also the sexist culture in work environments could explain the consistency of results showing a higher risk of workplace bullying among women. Regarding age and marital status, results largely vary according to the type of job. Possibly in certain types of jobs with a demand for rapid response or physical effort, such as in blue-collar jobs, older workers could be more likely to be bullied. Also, in a job requiring a flexible schedule, people with children could be the main targets. Despite the fact that studies usually show a higher vulnerability of black people and ethnic minorities for several exposures and health outcomes—such as racial discrimination [81]—very few studies evaluated the association between ethnicity and bullying and results on this matter are controversial.

Findings from four papers [26,40,48,76] are consistent with a systematic review [21], indicating a positive association between bullying and neuroticism. However, other personality traits were not associated with bullying. Effect sizes of the association between neuroticism and bullying were small, so residual confounding should be considered. The explanatory power of personality as a predictor of bullying was also very small [58]. One of the premises of occupational health actions is that the work environment and the work organization should be adequate to individual characteristics, not the opposite. Thus, interventions to prevent bullying focused on individual aspects tend to be limited. Notwithstanding, studies investigating the effect of work organization on bullying should evaluate these factors as confounders or effect modifiers.

The effect of sociodemographic, personality and some occupational factors on bullying varied across studies, while a poor work organization and poor working processes always increased the risk of bullying. This risk varies across professions because it depends much more on the work environment than the profession itself or the years of work. For example, since workers’ resignation is not common among civil servants, they can be easy targets of bullying, mainly when organizations are not prepared to support employees not well adapted to employers’ demands. Workers with lower income, paid hourly, and less satisfied with their payment, as well as those in shift work, are usually exposed to more precarious working conditions, making these individuals more vulnerable to bullying.

In a context of ‘flexible restructuring’ of capitalism [82], the fundamental role of the capital and labour conflict on the existence of workplace bullying is unveiled by the human resources management (HRM) ideology [80,83]. Workplace bullying plays an important role in the intensification of work processes, being a tool to deepen mechanisms to control workers [84]. As a ‘functional discipline’, HRM lies at the core of organizational design and practice, shaping the way organizations operate [83,85]. The importance of HRM as a main determinant of bullying is evidenced by the positive association between low moral standard, lack of procedural justice, organizational change, and low psychosocial safety climate with bullying. 

HRM ideology determines leadership styles in organizations and, agreeing with a systematic review about bullying in nurses [22], authoritarian and laissez-faire leadership patterns were strongly related to workplace bullying in this review [53,64,66,69,74] while ‘democratic’ leadership styles—such as supportive [57], authentic [69], transformational [66,69] and fair leadership [73]—protected the organization against bullying. Leaders are selected to put in practice the organizations’ ‘values’ and ‘missions’ and an authoritarian leadership pattern is still highly valued in several companies [86]. A poor work organization, where workers are under a lot of pressure and/or ethical values are secondary, is more likely to request authoritarian leadership. In some organizations, once its existence can improve productivity and accomplishments, bullying is institutionalized and works as an essential part of leadership and managerial practice [3,87]. This can also be one of the reasons why interventions against bullying are often ineffective [18].

Leadership patterns are strongly related to the work organization and might determine role conflicts, role ambiguity, flexibility in work methods, and unclarity of duties. All these problems in work methods and in management increase the risks of bullying, results that agree with former reviews [19,20]. These hazards are related to the post-Fordist models; with the restructuring of production, workers need to be ‘super-qualified’, polyvalent, and able to perform several tasks and activities [82,88]. These organizational aspects, while they intensify working processes, generate workloads such as work pressure, monotonous tasks, rotating tasks, high demands and stress, which also increase the risk of workplace bullying. On these aspects, a bidirectional association is also plausible, as the existence of bullying in an organization degrades the work environment [80,89] increasing workloads and intensifying working processes.

The findings from this review reinforce Leymann’s theoretical model, highlighting the central role of organizational factors on bullying determination. HRM, leadership patterns, and organizational factors are key distal determinants, having an impact on the physical and psychosocial workloads which determine bullying.

## 5. Conclusions

We have a large amount of valuable data concerning workplace bullying in high-income countries, particularly in Europe. However, a major effort is still necessary to encourage research on workplace bullying in low- and middle-income countries. Discrimination and harassment are more often described in non-dominant or disadvantaged groups [90], so a social context with poorer working conditions could lead to a higher risk of workplace bullying.

The main limitations of our review were the low response rates in most of the selected papers, as well as the variability of measures to assess risk factors and outcome, reducing the comparability of findings. Also, most of the studies were cross-sectional and were not able to estimate the effect of all occupational factors on bullying, precluding strong inferences regarding the direction of associations. Therefore, future studies with a longitudinal design and representative samples (or at least a better description of losses) are important to clarify associations subjected to reverse causation and improve the interpretation of the results. It is necessary to deepen the understanding of the role of organizational factors and emphasize the role of human resource management on bullying causation. The effect of work schedules, breaks, and extra hours on bullying also need to be studied. Validated instruments, such as the Psychosocial Safety Climate-12, which addresses aspects of the management in work environments, can be fundamental to evaluate distal contextual factors. Considering the scarcity of information about the association between race and sexual orientation with bullying, future studies should also investigate this subject.

Bullying should be understood as a completely unacceptable and unethical behaviour in workplaces. Policies to prevent bullying must address the culture of organizations and face the challenge of developing psychosocial safety at work environments. Interventions promoting a new management and leadership framework, increasing democratic values, and promoting employee participation in work decisions, should be implemented and evaluated in order to provide better parameters for practice in occupational health.

## Figures and Tables

**Table 1:** Epidemiological studies about risk factors for workplace bullying.

N	Study/Year/Local	Study Population/ Sample	Study Design/Methods to Measure Bullying/Statistics	Confounders/ Adjust Variables	Main Results				Downs and Black Score
1	Rosta [26] 2018 Norway	Representative sample of Norwegian doctors. Response rates were 72.8% (2628/3608) in 1993, 67% (1004/1499) in 2004, and 78.2% (1261/1612) in 2014–2015. 485 doctors responded both in 2004 and 2014–2015.	Cross-sectional from three surveys Single question: ‘Have you during the last year been subjected to vexation or uncomfortable teasing (bullying) from colleagues or superiors?’ Logistic regression	Gender and age	Controlled for gender and age, neuroticism was a significant predictor in the cross-sectional samples from 2004 (odds ratio (OR) 1.28, 95% CI 1.13 to 1.44) and 2014–2015 (1.24, 95% CI 1.07 to 1.45). Introversion– extraversion showed no effect. Being a woman (OR = 2.02 (1.18 to 3.47)), having lower job satisfaction (OR = 0.92 (0.90 to 0.94)), and lower levels of self-rated health (good compared to very good (OR = 3.50 (1.49 to 8.25)) and average or poor compared to very good (OR = 2.29 (1.21 to 4.33))) were significant univariate and multivariate predictors of bullying.				14
2	Tong [57] 2017 Switzerland	5311 care workers from 162 randomly selected nursing homes with 20 or more beds in Switzerland. Response rate = 76%. Sub-study of the Swiss Nursing Homes Human Resource Project (SHURP).	Cross-sectional, multi-centre study. National Acts Questionnaire (NAQ)-short version Generalized estimation equation models with binary logistic regression	Facility characteristics (size, profit status, language region) Workers characteristics (gender, age, educational background, length of employment in nursing home, and percentage employment).	Supportive leadership style OR = 0.42 (0.30–0.58) Staffing and resources adequacy OR = 0.66 (0.48–0.92) Teamwork and safety climate OR = 0.41 (0.30–0.58) Mobbing was associated with: ✓ intention to leave (OR: 5.12; 3.81–7.88) ✓ job satisfaction (OR: 0.19; 0.14–0.26) ✓ suffering from health issues (OR: 7.81; 5.56–10.96)				13
3	Amponsah-Tawiah [58] 2017 Ghana	Convenience sample from diverse occupations in Ghana. 750 questionnaires distributed, 631 were returned (response rate = 84%).	Cross-sectional NAQ-r Hierarchical regression model including demographic characteristics (1st level), personality characteristics (2nd level), and organizational politics (3rd level).	Age, sex, marital status, level of education, and position in organization	The overall model was significant and accounted for 22% of the variance in workplace victimization (adjusted R^2^ = 0.22). Model including demographic and personality characteristics explained only 2% of the variance (adjusted R^2^ = 0.02), while model including only organizational politics explained 19% of the variance (adjusted R^2^ = 0.19) Personality was significantly associated with workplace bullying in a small magnitude, whereas organizational politics were positively and strongly related to workplace bullying.				8
4	Pihl [59] 2017 Denmark	Representative sample of the Danish working population (*n* = 10,037). Response rate = 53%.	Cross-sectional Single question: ‘Have you within the last 12 months been exposed to bullying at your workplace (i.e., over several months been exposed to unpleasant or humiliating acts which have been difficult to defend yourself against)?’ Logistic regression	Age, sex, seniority, work environment variables, and work-related self-efficacy	Low and medium social capital (vertical) are strongly associated to bullying: OR = 3.25 (2.34–4.51) (low)/OR=1.59 (1.16–2.18) (medium) Low social capital (horizontal) is strongly associated with bullying: OR = 3.17 (2.41–4.18) (low)				13
5	Norton [39] 2017 Portugal	Of the 5657 questionnaires provided to workers at the São João Hospital Centre (SJHC), the first 707 returned were included in this study. Response rate = 12.5%	Cross-sectional NAQ Chi-squared and logistic regression	Gender, age group, occupational group, type of contract, and work schedule	After adjustment, only one type of contract (indefinite duration employment contracts) was associated with workplace bullying; OR= 0.43 (0.20-0.95) (government employees were the reference group). OR showed wide confidence intervals				9
6	Guglielmi [60] 2017 Spain	Sample of 195 Spanish employees from different occupational sectors filled in an online questionnaire at two different times with a time lag of eight months.	Longitudinal study NAQ Moderated mediation model based on 5000 bootstrap re-samples	Gender and job tenure	The index of moderated mediation was significant: *B* = −0.120, SE = 0.061, 95% CI (−0.259; −0.014). Analysis revealed a conditional indirect effect of T1 Effort-Reward Imbalance (ERI) on T2 workplace bullying through T2 organizational justice, with the indirect effect significant at low (−1SD; *B* = 0.383, SE = 0.104, 95% CI (0.214; 0.626)) and moderate (mean; *B* = 0.267, SE = 0.088, 95% CI (0.119; 0.455)) levels of T1 organizational identification. There was also a direct effect of Effort-Reward Imbalance (ERI) on workplace bullying; *B* = 0.456 (0.134; 0.778), *p* < 0.01.				7
7	Forsell [27] 2017 Sweden	1,972 (10% women, 90% men) seafarers with a personal e-mail address in the Swedish Maritime Registry (5608 e-mails were sent). Response rate = 35%	Cross-sectional Single question: ‘Have you at least once during the last 12 months felt exposed to offensive actions or harassment at your work place?’ For example—your actions or comments were ignored, you are not taken seriously, were ridiculed or patronized (y/*n*). T-test and chi-squared	Age	Although common among men (22%), offensive actions or harassment were twice as common in women (45%; PR 2.0; 95% CI 1.6–2.4, controlling for age). The majority of female engine room crew members reported harassment or bullying, but they were few in total numbers (11/19; 58%).				10
8	Fernandez [52] 2017 United States	14,725 individuals from probability household national survey: 2010 National Health Interview Survey (NHIS) Occupational Supplement and 2010 Occupational Information Network online (O*NET) database.	Cross-sectional ‘Workplace harassment’ was defined as participants answering ‘yes’ to the question: ‘During the past 12 months, were you threatened, bullied, or harassed by anyone while you were on the job?’ Multivariable logistic regression	Age, race, education, and type of work.	Being a green-collar worker: OR = 0.77 (0.62–0.95) (reference: non-green-collar) Having an older age (>65 years): OR = 0.37 (0.22–0.64) (reference: 19–44 years) Race (others) 0.72 (0.54–0.96) (reference: white) Education was not associated with harassment.				8
9	Bayramoglu [55] 2017 Turkey	1189 forest engineers working at 25 different Regional Directorates of Forestry in Turkey.	Cross-sectional NAQ-r (analysed in three outcomes) T-test, Analyses of Variance (ANOVA), multinomial regression analyses	Gender, in house position, age, marital status, educational level, duration of professional life.	Three categories of bullying: relevant to person (RP); tasks related (TR); physical violence/verbal threat (PV/VT) RP was associated with age, duration of professional life, and type of leadership. TR was associated with gender, age, duration of professional life, and type of leadership. PV/VT was associated with educational level.				6
10	Rouse [31] 2016 United States	Part of the larger Council of Academic Family Medicine Educational Research Alliance omnibus Survey. 1049 individuals (33.5% response rate) from 3184 academic family physicians.	Cross-sectional NAQ-r + direct questions Chi-squared	None	Prevalence of being bullied: Women = 34.0% answered ‘yes’ (150/441) Men = 24.7% answered ‘yes’ (139/563). (*p* < 0.001) Prevalence of being a perpetrator: Women = 7.7% Men = 11.2%				6
11	Medina-Gomez [40] 2016 Mexico	499 workers who attended one medical unit.	Cross-sectional Inventario de violencia y acoso psicológico em el trabajo (IVAPT-Pando) Poisson regression	Age	ORs (Risk factors for bullying): Female Sex (compared to male) = 1.07 (0.92–1.23) Neuroticism (compared to stability) = 1.23 (1.04–1.46) Self-satisfaction (compared to high satisfaction) ✓ Average 1.61 (1.33–1.93) ✓ Low 1.147 (1.20–1.79) Depersonalisation (compared to none) High = 2.08 (1.64–2.64)				8
12	Gardner [61] 2016 New Zealand	826 workers from New Zealand. Does not describe whether they were randomly selected. Time 1: 991 men (40.9%) and 1421 women (58.6%). Time 2: 349 men (42%) and 477 women (58%).	Cohort, two waves, three months apart NAQ-r Regression and correlational analysis	Gender Role Performance Absenteeism Physical health Strain Ethical leadership Destructive leadership Perceived organizational support (POS) Team conflict Effectiveness of org. responses	Job performance and absenteeism were unrelated to workplace bullying. Those with worse physical health (beta = 0.15, *p* < 001) and higher strain (beta = 0.11, *p* < 0.05) at Time 1 experienced more bullying at Time 2. There was stronger support for the importance of organizational factors in workplace bullying. While positive organizational resources, such as ethical leadership and POS, were not related to workplace bullying, destructive leadership (beta = 0.22, *p* < 0.001) and more team conflict (beta = 0.20, *p* < 0.001) at Time 1 were associated with higher levels of bullying at the Time 2. Effective organizational strategies were protective (beta = −0.11, *p* < 0.01). Full model explained 37% of bullying variance.				12
13	Ariza-Montes [62] 2016 Spain	5th European Working Conditions Survey, including 27 European countries. Sub-sample of 261 employees (48.7% experiencing workplace bullying) from 2873 teaching professionals.	Cross-sectional Single question: ‘Over the past 12 months, during the course of your work, have you been subjected to bullying/harassment?’ Structural equation model	Not described.	Stress and motivation explained 11.2% of workplace bullying. Six causation hypotheses were tested between job demands (JD), job resources (JR), stress (S), motivation (M), and workplace bullying (WB). H1: JD→S 0.315 (2.118) *p* < 0.05 H2: JD→M (−0.177) (1.274) ns H3: JR→M (0.416) (4.167) *p* < 0.001 H4: JR→S (−0.104) (0.739) ns H5: S→WB 0.245 (4.191) *p* < 0.001 H6: M→WB _0.218 (4.011) *p* < 0.001 Job demands were associated with stress. Job resources were associated with motivation. Stress and motivation were strongly associated with workplace bullying, supporting the work environment hypothesis.				12
14	Tsuno [63] 2015 Japan	5000 workers randomly selected, 2384 participants. Response rate = 47.7%. After excluding 87 with missing data and 751 who were not active in the labour force at that time, the final sample was 1546 respondents. (809 men and 737 women), aged 20–60 years old.	Cross-sectional Bullying assessed using a single question: ‘Have you been bullied in your workplace during the past 30 days?’ Multiple logistic regression	Gender and age Full model included education, household income, occupation, employment contract, company size, establishment size, and type of industry.	After adjusting for gender and age: Temporary employees OR = 2.45 (1.03–5.85) Junior high school graduates OR: 2.62 (1.01–6.79) Workers with lowest household income OR: 4.13 (1.58–10.8) Workers in the lowest subjective social status (SSS) stratum OR: 4.21 (1.66–10.7)				14
15	Tsuno [64] 2015 Japan	All civil servants in the city (n = 2069) located in the east coast region of Japan. 99 questionnaires were returned. 404 participants also returned follow-up questionnaire (response rate of 40.8%). After 87 exclusions (missing values), 317 workers were analysed.	Cohort, 6-months follow-up NAQ-r, Leymann criteria Multiple logistic regression	Gender, age, education, marital status, chronic condition, occupation, employment contract, shift work at baseline and life events in the previous six months at follow-up. Full model included leadership characteristics.	Passive laissez-faire leadership increased 4.3 times (95% CI: 1.29−14.2) the risk of new exposureto bullying, (*p* for trend = 0.018). Respondents whose supervisors had high consideration of the individual had a 70% lower risk of new exposure to bullying. The results of a multilevel analysis showed that group level charismatic/inspirational leadership, intellectual stimulation leadership, individual consideration leadership, and contingent reward leadership had significant negative relationships with individual follower experiences for workplace bullying (γ = −4.02, −3.12, −3.41, and −3.63, all *p* < 0.05). On the other hand, passive laissez-faire leadership had significant positive relationships with individual follower experiences for workplace bullying (γ = 4.29, *p* < 0.01).				15
16	Rodriguez-Muñoz [65] 2015 Spain	Stratified random sampling from 17 autonomous communities of Spain. 1000 employees were invited to participate and 600 (response rate = 60%) agreed to participate at the time 1 At the time 2 all 600 employees were invited to answer the same telephone interview, and 348 participated (response rate 58%).	Cohort: two-wave longitudinal study Time-lag: six months s-NAQ (9 items) Structural equation models	Gender and educational level	Time 1 (T1) vigor was negatively related to Time 2 (T2) workplace bullying (*β* = −0.18, *p* < 0.01), whereas T1 anxiety (*β* = 0.12, *p* < 0.05) was positively related to T2 workplace bullying.				15
17	Picakciefe [42] 2015 Turkey	119 from 130 healthcare workers from the city centre of Mugla, Turkey (91.5% response rate).	Cross-sectional Self-report of 28 types of mobbing behaviours based on Leymann’s conceptual framework Logistic regression	Gender, age, educational level, marital status, total working time, psychosocial reactions, and behaviours.	Marital status (married): OR = 3.06 (1.41–12.94) *p* = 0.024. Total working time (year: ≥16): OR = 2.72 (1.19–6.21) *p* = 0.018. Psychosocial reactions (yes): OR = 9.77 (4.72–25.53) *p* < 0.001 2. Counterproductive behaviours (yes): OR = 3.24 (2.50–29.39) *p* < 0.001.				14
18	Lipscomb [50] 2015 United States	11,874 participants from four agencies from unionized public-sector workforce in United States. Overall response rate (for three agencies) was 71.8% (61.5% to 81.9%). The fourth agency had an estimated response rate of 55–60%.	Cross-sectional NAQ + single question Chi-squared	None	Prevalence of bullying was higher in: Men 2.4 vs 2.1 women (*p* < 0.01). Non-white 3.0 vs. 2.3 white (*p* < 0.05). Age 36–45 2.6% vs. 2.1 age <36 (*p* < 0.05). Support/administrative workers 2.6% vs. professionals 2.0% vs. management/confidential 0.7% (*p* < 0.01).				9
19	Dussault [66] 2015 Canada	Sample of 288 adults 153 were attending evening undergraduate classes in organizational behaviour management at a Canadian regional university, and 135 were employed within a multinational company in construction.	Cross-sectional NAQ-r Structural Equation Modelling. Given the non-normality of the data, robust maximum likelihood estimation was used.	Covariates not described.	Transformational leadership was negatively related to work-related bullying (β = –0.57), perceived Person-related bullying (β = –0.57), and perceived physically intimidating bullying (β = –0.45). Transactional leadership was also negatively related to work-related bullying (β = –0.38), perceived Person-related bullying (β = –0.30), and perceived physically intimidating bullying (β = –0.14). Laissez-faire leadership was positively related to work-related bullying (β = 0.51), perceived Person-related bullying (β = 0.53), and perceived physically intimidating bullying (β = 0.51).				6
20	Ariza-Montes [33] 2015 Spain	5th European Working Conditions Survey, including 27 European countries	Cross-sectional Single question: ‘Over the past 12 months, during the course of your work, have you been subjected to bullying/harassment? Logistic regression	Sex, age, having children at home, working hours, night shift, type of contract, shift work, working day, responsibility, complex tasks, motivation, likely to be dismissed, flexibility, expectation of career growth, work stress, working condition satisfaction, wage satisfaction, and company size.	Public sector: The best predictors of workplace bullying were: working condition satisfaction (odds ratio (OR), 3.04; CI, 1.93 to 4.80), shift work (OR, 2.46; CI, 1.53 to 3.95), motivation (OR, 2.14; CI, 1.52 to 3.02), work stress (OR, 2.40; CI, 1.60 to 3.60), flexibility in work methods (OR, 1.93; CI, 1.32 to 2.82), and gender (female) (OR, 1.81; CI, 1.28 to 2.56). Private sector: The best predictors were: satisfaction with working conditions (OR, 4.37; CI, 3.11 to 6.15), work stress (OR, 2.01; CI, 1.45 to 2.79), shift work (OR, 1.94; CI, 1.37 to 2.73), gender (female) (OR, 1.78; CI, 1.35 to 2.36), satisfaction with the wage perceived (OR, 1.77; CI, 1.32 to 2.37), and type of contract (OR, 1.74; CI, 1.19 to 2.56).				13
21	Tuckey [67] 2014 Australia	Retail workers (n = 4000) identified from the Shop Distributive and Allied Employees’ Association (SDA) South Australian membership database were invited to participate in a self-report survey. A total of 609 responded at Time 1 (response rate = 15%), and 419 at Time 2. Final sample: 221 participants who responded at both waves (36% of the original Time 1 sample).	Cohort: two waves, six months apart 10 items relevant to retail work from a short version of the NAQ. Structural equation modelling	Not described.	The ‘path’ from emotional exhaustion (Time 1) to workplace bullying (Time 2) was significant (beta = 0.14, *p* < 0.05)				10
22	Salin [32] 2014 Finland	Representative sample of Finnish employees (n = 4392).	Cross-sectional Single question Logistic regression	Gender, age, leadership, job demands, physical work environment, gender incongruence, and performance-based pay.	Men (OR = 0.676, CI = 0.550–0.831) and older employees (OR = 0.988, CI = 0.979–0.997) reported a significantly lower risk of having observed bullying in their work communities. High job demands (OR = 2.001; CI = 1.620–2.471), constructive leadership (OR = 0.776, CI = 0.688–0.902), and a poor physical work environment (OR = 1.430, CI = 1.238–1.651) were associated with bullying. Gender-congruence of the respondent’s work tasks and the compensation system were not related to observations of bullying.				11
23	Reknes [68] 2014 Norway	2835 Norwegian employees, from 20 Norwegian organizations in the private and public sectors, collected during the period 2004 to 2009.	Cohort Single question Logistic regression	Gender, age, and educational level	Role ambiguity OR = 1.58 (1.18–2.13) Role conflict OR = 1.92 (1.43–2.57)				12
24	Khubchandani [34] 2014 United States	National Health Interview Survey (NHIS) 2010 data A total of 17,524 adults were included in this study (51.5% females, 74.9% white, 46.3% married, and 73.3% worked for a private company).	Cross-sectional Single question: ‘’During the past 12 months, were you threatened, bullied or harassed by anyone while you were on the job?’ Logistic regression	None regarding descriptive data.	Prevalence of harassment = 8.1% Odds was higher in: Females OR = 1.47, *p* < 0.001 Multiracial individuals OR = 2.30, *p* < 0.001 Divorced or separated individuals OR = 1.88, *p* < 0.001 Individuals who worked for the state OR = 1.74 (1.28–2.37), *p* < 0.001 Individuals who worked for state local government: 1.73 (1.30–2.30), *p* < 0.001 Regular night shifts OR = 1.74 (1.16–2.62), *p* < 0.01 People who have more than one job OR = 1.38 (1.01–1.94), *p* < 0.01 Paid hourly OR = 1.30 (1.10–1.55), *p* < 0.001				13
25	Ariza-Montes [35] 2014 Spain	Sample population of 661 Managers was obtained from the micro data file of the 5th European Working Conditions Survey 2010	Cross-sectional Single question: ‘Over the past 12 months, during the course of your work have you been subjected to bullying/harassment?’ Logistic regression	Gender, having children at home, work at night, shift work, work stress, satisfaction with work, satisfaction with payment, and opportunities for promotion	The risk for a manager to feel bullied was higher in: ✓ women (OR: 1.72; CI: 1.09–2.72) ✓ workers with small children at home (OR: 1.92; CI: 1.22–3.02) ✓ those who work at night (OR: 2.02; CI: 1.17–3.48) ✓ those on a shift system (OR: 1.95; CI: 1.03–3.67) ✓ those who suffer from work stress (OR: 4.65; CI: 2.43–8.86) ✓ those who feel little satisfied with their working conditions (OR: 3.54; CI: 1.91–6.56) ✓ those less satisfied with their payment (OR: 2.90; CI: 1.85–4.56) ✓ those who do not see opportunities for promotion within their organizations (OR: 1.72; CI: 1.08–2.73)				14
26	Toksoy [37] 2013 Turkey	27 Regional Directorates of Forestry that are under the aegis of the Ministry of Forestry and Water Affairs. The questionnaire was filled in by 845 forest engineers.	Cross-sectional NAQ-r (analysed in four factors) T-test and ANOVA	ANOVA analyses included: Age Marital status Education level Duration of the professional life Change of the number of units worked Geographical location	Female forest engineers were more exposed to humiliation compared to male (*p* ≤ 0.05), People in the 34–44 age group were more exposed to ‘relevant to person’ (*p* ≤ 0.05) and ‘task-related’ behaviours (*p* ≤ 0.05). No significant relationship was found between the marital status and the levels of exposure to bullying. A significant relationship was found between education level and humiliation (*p* ≤ 0.05), indicating that forest engineers with a doctor’s degree were more exposed to humiliation compared to those with a bachelor’s or a master’s degree.				6
27	Nielsen [69]) 2013) Norway	594 seafarers working on 40 vessels from two large Norwegian shipping companies. Response rate = 73% of 817 crew members working at that time.	Cross-sectional NAQ-r Binary logistic regression and mediation analyses	Age Bullying behaviours Laissez-faire leadership Transformational leadership Authentic leadership Group cohesion Safety perceptions	Type of leadership and occurrence of bullying: Laissez-faire leadership - OR = 3.25 (2.21–4.79) Transformational leadership - OR = 0.58 (0.36–0.94) Authentic leadership OR = 0.50 (0.33–0.78)				13
28	Carter [51] 2013 United Kingdom	2689 staff from seven NHS Trusts from the northeast of England. Convenience sample (1.2% to 22.2% response rate depending on the occupational group).	Cross-sectional NAQ-r (mean scores) Multivariate Analysis of Variance (MANOVA)	Not described for data of interest.	There was no significant difference on the overall NAQ-R mean score between white (27.3) and black or ethnic minority staff (27.5), t(2546) = 0.26, *p* = 0.80 The overall NAQ-R mean score was significantly higher for male staff (28.3) than female staff (27.0), t(925.4) = 3.15, *p* = 0.002.				7
29	Carretero [43] 2013 Spain	422 workers from 61 centres answered Time 1 and Time 2 (response rate of T1 sample = 61.82%).) At T1, 1470 questionnaires were distributed in 66 care centres for people with intellectual disability in Valencia. T1 response rate = 47.32% (696 workers).	Cohort Mobbing-UNIPSICO Questionnaire. This scale contains 20 items adapted from the Leymann Inventory of Psychological Terrorization (LIPT) and the Negative Acts Questionnaire (NAQ) Chi-squared and Student’s t-test	None	At Time 1, no statistically significant differences between victims and non-victims were found with respect to gender, age, civil status, and years of service in the profession. Statistically significant differences were found between workplace bullying victims and non-victims in contract type (*p* < 0.05), in years with the organization (*p* = 0.004), and in the position (*p* = 0.006), indicating that a higher percentage of workplace bullying victims have a stable contract, more years of service, and a longer period in the position. At Time 2, no statistically significant differences between victims and non-victims were found by gender, age, marital status, contract, and years of service in the profession, in the organization or in the position.				7
30	Ariza-Montes [36] 2013 Spain	Sub-sample of 284 health professionals 5th European Working Conditions Survey 2010.	Cross-sectional Single question: ‘Over the past 12 months, during the course of your work, have you been subjected to bullying/harassment?’ Logistic regression	Gender, age, education, children at home, and occupational characteristics	Risk Factors:	*p*-value	OR	95% CI	13
					Gender (0: male; 1: female)	0.005	2.77	(1.36–5.66)	
					Age (0: 15–24; 1: 25–39; 2: 40–54; 3: 55 or over)	0.065	0.63	(0.38–1.03)	
					Level of education (0: university education, 1: secondary education)	0.003	5.51	(1.79–16.95)	
					Children at home (0: yes; 1: no)	0.014	2.87	(1.24–6.63)	
					Shift work (0: no; 1: yes)	0.005	2.68	(1.35–5.31)	
					Monotonous tasks (0: no; 1: yes)	0.025	2.20	(1.10–4.40)	
					Rotating tasks (0: no; 1: yes)	0.010	2.60	(1.26–5.39)	
					Work stress (0: no; 1: yes)	0.003	4.96	(1.70–14.46)	
					Working condition satisfaction (0: yes; 1: no)	0.033	2.43	(1.07–5.51)	
					Expectation of career growth (0: yes; 1: no)	0.000	4.52	(2.09–9.76)	
31	Oxenstierna [53] 2012 Sweden	Swedish occupational longitudinal study of health 2203 individuals.	Cohort Single question: “Are you exposed to personal persecution by means of vicious words or actions from your superiors or your workmates?” Multiple logistic regressions	Age, education, sector, supervisory duties, and all workplace characteristics	Sociodemographic factors: Age in men was associated with bullying (OR = 0.74; 0.55–0.99) No association with age in women. No association with educational level and work sector. Organizational factors: Dictatorial leadership in men (OR = 1.79; 1,29–2.49), organizational change in women (OR = 1.28; 1.00–1.63), lack of procedural justice in men (OR = 1.54;1.00–2.38) and social support, lack of humanity in women (OR = 1.61; 1.10–2.35), and attitude of expendability in men (OR =1.59; 1.13–2.23) were associated with bullying. Conflicting demands in men (OR = 1.52; 1.14–2.04) and decision authority in women (OR = 0.77; 0.61–0.97) were associated with bullying.				12
32	Figueiredo-Ferraz [70] 2012 Spain	422 Spanish employees working with people with intellectual disabilities at 61 companies. in the Valencian community. Response rate = 61.82%.	Cross-sectional Mobbing-UNIPSICO scale (20 items adapted from the LIPT and from the NAQ) Structural equation model	Not described	The relationships between role clarity (coef = −0.19, *p* < 0.001), interpersonal conflict (coef = 0.27, *p* < 0.001), social support at work (coef = −0.32, *p* < 0.001), and mobbing were significant and in the expected direction. Role clarity, interpersonal conflict, and social support at work explained 37% of the variance of mobbing.				12
33	Sahin [56] 2012 Turkey	278 male physicians who started compulsory military service in the Ministry of Defence in April 2009 Response rate: 95%.	Cross-sectional LIPT Structural equation model	Sociodemographic) characteristics: ✓ work place ✓ marital status ✓ specialty status ✓ number of working hours per week ✓ age ✓ duration of work (years) ✓ occupational commitment ✓ personality Five variables: ✓ behaviour threatening communication ✓ behaviour threatening social contacts ✓ behaviour threatening personal reputation ✓ behaviour threatening occupational situation ✓ behaviour threatening physical health	Four factors had significant effects on mobbing: ✓ working place coef −0.132, *p* = 0.027 ✓ marital status coef −0.132, *p* = 0.027 ✓ weekly working hours coef −0.252, *p* < 0.01 ✓ occupational commitment coef −0.141, *p* = 0.018 ✓ They explained 12% of variance.				13
34	Askew [44] 2012 Australia	747 participants of the Australian medical workforce DeC Study Convenience sample	Cross-sectional Single question: ‘In the last 12 months, have you been subjected to persistent behaviour by others which has eroded your professional confidence or self-esteem?’ T-test, Fisher exact test	None	There were no differences in the reported rates of bullying across age groups, sex, and country of medical qualification.				6
35	Notelaers [45] 2011 Belgium	8985 Flemish speaking respondents within 86 firms spread over the main sectors of Flemish working life.	Cross-sectional NAQ (Belgium version) Polinomial regression	Gender, age, occupational status, sector, employment contract, working hours.	Employees at higher risk of being a victim of bullying: ✓ age of 35 and 45 OR = 1.74 ✓ age of 45 to 54 OR = 1.92 ✓ public servants OR = 4.78 ✓ blue-collar workers OR = 2.16 ✓ from the manufacturing industry OR = 1.93 ✓ from the food industry OR = 3.34 ✓ Gender was not associated with the odds of bullying. Temporary contract and other professions were not associated with bullying. Working schedule was associated with being bullied sometimes.				12
36	Law [71] 2011 Australia	215 Australian income earners from randomly selected households from the state of South Australia. The overall sample response rate was 31.2% and the participation rate was 38.4% of 1134 participants who completed the Australian Workplace Barometer Questionnaire (AWBQ2009).	Cross-sectional Single question: ‘Have you been subjected to bullying at the workplace during the last 6 months?’ ANOVA and multilevel mediation analysis	Age, gender, and income	The relationship between organizational PSC and bullying/harassment was negative and significant, B = −0.25, S.E. = 0.06, t = −3.51, *p* < 0.01.				13
37	Keuskamp [46] 2011 Australia	Initial sample of 4500 households, 3103 in-frame contacts, 1853 households were surveyed (response rate = 59.7%). A total of 1016 self-reported as currently employed.	Cross-sectional Single question: ‘Have you personally experienced bullying in your current job?’ Chi-squared and logistic regression	Age, sex, marital status	Bivariate analysis: Prevalence of bullying was higher in: ✓ Permanent workers 19.6% vs. 7.7% casual workers ✓ Separated, divorced, widowed 28.8% vs. married/de facto 12.2% vs. never married 14.6% No differences between sex, educational level, and financial status After controlling (logistic regression model), only marital status remained associated with bullying (OR = 2.26 (1.28–3.99))				14
38	Carretero-Dominguez [72] 2011 Spain	T1 = 696 participants from 1470 questionnaires distributed in 66 assistance centres (47.3% response rate) T2 = 422 participants (61.8% response rate).	Cohort Mobbing-UNIPSICO (based on LIPT and NAQ) Structural equation model	Interpersonal conflicts, role conflicts, role ambiguity, social support	In cross-sectional analyses (T1 and T2), all factors (interpersonal conflicts, role conflicts, role ambiguity and social support) were associated with workplace bullying. ✓ interpersonal conflicts (T1 coef = 0.17, *p* < 0.05; T2 coef = 0.03, *p* = ns) ✓ role conflicts (T1coef = 0.34, *p* < 0.05; T2 coef = 0.36, *p* < 0.05) ✓ role ambiguity (T1 coef = 0.08, *p* = ns; T2 coef = 0.14, *p* < 0.05) ✓ social support (T1 coef = −0.26, *p* < 0.05; T2 coef = −0.26, *p* < 0.05) In longitudinal analyses, only social support was associated with bullying (coef = −0.09, *p* < 0.05). Variables explained 52% of mobbing.				10
39	Trijueque [38] 2009 Spain	2861 workers from several workforce sectors (4000 questionnaires distributed).	Cross-sectional NAQ-r Chi-squared	None Analyses were descriptive.	Groups more likely to being bullied: Female (6.9% vs. 4.3%) male Public sector (9.1% vs. 4.8%) Private Companies with less than 50 employees (6.1% vs. 5.4%) >50 employees Unionized (9.9% vs. 5.1%) non-unionized Sick leave (current and previous) Treatment (current and previous)				7
40	Ortega [47] 2009 Denmark	3429 employees between 20 and 59 years from the second Danish Psychosocial Work Environment Study (DPWES). Response rate = 60.4%.	Cross-sectional Bullying was assessed with a single question: ‘Have you been bullied in the past 12 months?’ Chi-squared	None	No significant gender or age differences were found. Unskilled workers reported the highest prevalence of bullying (13.5%), while managers/supervisors the lowest prevalence (4%). People working with things (male-dominated occupations) and people working with clients/ patients (female-dominated occupations) reported higher prevalence of bullying than people working with symbols or customers.				10
41	Mageroy [73] 2009 Norway	1604 military personnel from the Royal Norwegian Navy were included in the analyses. Response rate = 62.5% (1657 of 2652).	Cross-sectional Single question: ‘Have you been subjected to bullying or harassment at the workplace during the last six months?’ Logistic regression	Age and sex	Fair leadership, OR = 0.59 (0.44–0.78) Innovative climate, OR = 0.71 (0.52–0.96) Inequality, OR = 0.72 (0.60–0.86) Empowering leadership, OR = 1.36 (1.07–1.73) Human resource primacy, OR = 0.77 (0.60–1.01) Support from superior, non significant (ns) Support from co-workers, ns Support from friends and relatives, ns Main organizational categories: Defence command and other offices vs. operational, OR = 0.42 (0.24–0.75) Logistics vs. operational, OR = 0.60 (0.35–1.02), ns Schools vs. operational, OR = 1.18 (0.80–1.76), ns				15
42	Agervold [74] 2009 Denmark	898 participants from 12 different local government social security offices (local authority-educated social workers with equivalent competence and general office personnel). 1023 questionnaires were distributed. Response rate = 88%.	Cross-sectional NAQ (10 negative acts) Chi-squared and Mann–Whitney	None	Demands of work, pressure of work, autocratic management style, unclarity of duties, and social work climate were strongly associated with bullying.				12
43	Matthiesen [75] 2008 Norway	4742 participants from six Norwegian labour unions and members of the Norwegian Employers’ Federation (NHO) from a total population of 10,616 individuals. Response rate = 47%.	Cross-sectional Single question: ‘Have you been subjected to bullying at the work place during the last six months?’ ANOVA	None.	Lack of self-esteem and social competency were positively associated with bullying. Role conflict and role ambiguity were positively associated with bullying.				12
44	Niedhammer [28] 2007 France	7770 respondents from 19,655 employees from the general working population in the southeast of France. National Institute of Health and Medical Research (INSERM) in 2004 Response rate = 40%.	Cross-sectional LIPT + single question Chi-squared and Logistic regression	Adjusted for age Stratified by gender	A total of 343 men (10.95%) and 583 women (12.78%) had experienced bullying weekly or more, and for 6 months or more (Leymann’s definition). Using self-reported exposure, 684 men (21.84%) and 1223 women (26.81%) reported being exposed to bullying within the last 12 months. Using both Leymann’s definition and self-reported exposure, 275 men (8.78%) and 488 women (10.70%) had been bullied. For men, the point prevalence was significantly higher among services activities, and lower among managers and professionals. For women, no significant difference was found according to economic activities and occupations. For men, the point prevalence ranged from 3.69% in construction to 14.63% in other community, social and personal service activities, and from 3.27% in physical, mathematical, and engineering science professionals to 17.74% in protective services workers.				12
45	Glaso [76] 2007 Norway	144 total participants, 72 bullied and 72 not bullied (matched control group regarding demographic variables; work tasks, age and gender).	Cross-sectional NAQ T-test	None (used a matched control group)	There were significant differences between victims and non-victims on four out of five personality dimensions. Victims tended to be more neurotic and less agreeable, conscientious and extraverted than non-victims. However, a cluster analysis showed that the victim sample can be divided into two personality groups. One cluster (64% of the victims sample), did not differ from non-victims. On the other hand, a small cluster of victims tended to be less extrovert, less agreeable, less conscientious, and less open to experience but more emotionally unstable than victims in the major cluster and in the control group.				9
46	Pranjic [48] 2006 Croatia	511 physicians from 1 hospital and 7 health centres in Tuzla, Brčko District and Banja Luka region. Response rate = 73% (total of 700 in the target population).	Cross-sectionalMobbing questionnaire (produced by researchers)Chi-squared	None	Explicitly type A personality (people with a chronic sense of time urgency, usually busy and very competitive, even in non-competitive situations) was the only factor associated with the bullying report. Age, gender, hours of work, and job title were not associated with the bullying report.				11
47	Bilgel [49] 2006 Turkey	877 full-time government employees in the three main public sectors: health, education, and security. 25 primary healthcare units and one public hospital, nine schools (two kindergartens, four primary schools, three high schools) and 13 police stations were randomly selected. Final response rate = 73.0%	Cross-sectional 20-item inventory of bullying developed by Quine Logistic regression	Gender, age, marital status, occupational characteristics	Occupation (doctors) OR = 0.34 (0.09–0.93), *p* = 0.035 (reference: secretary) Low support at work OR = 3.02 (2.22–4.11), *p* < 0.001 High stress OR = 1.38 (1.15–1.66), *p* = 0.001 Low job satisfaction OR = 1.98 (1.46–2.68) *p* < 0.001 Gender, age, marital status, work sector, and working years were not associated to bullying.				13
48	Varhama [54] 2004 Finland	1979 permanent employees from a municipality in Finland. A total of 3500 questionnaires were distributed. Response rate = 56.5%	Cross-sectional Single question Kruskall–Wallis	None	Prevalence of bullying increased with age, being higher in those aged 50–62 years old, followed by 40–49, 29–38, and 18–28, respectively. Prevalence of bullying was higher in the Fire Department, compared to Technical, Educational, Health, and Social Departments.				11
49	Quine [29] 2002 United Kingdom	594 junior doctors from 1000 randomly selected from the BMA members’ mailing list. Response rate = 62%, excluding 48 questionnaires that returned undelivered by the post office.	Cross-sectional Previous definition and a single question whether the person had been subjected to bullying in the past 12 months. Also, a 21-item bullying scale. Chi-squared	None	Black and Asian doctors were more likely to report being bullied than white doctors (78 (45%) vs. 139 (34%); RR = 1.59 (1.11–2.28). Women were more likely to report being bullied than men (43% (126) vs. 32% (92); RR = 1.61 (1.14–2.26). Reports of bullying did not vary by job grade or age.				10
50	Quine [41] 1999 United Kingdom	1100 out of 1580 employees from a community NHS trust in southeast England, as part of a larger survey of working life in 1996 Response rate = 70%.	Cross-sectional Scale with twenty types of bullying behaviour were taken from the literature, based on Rayner and Hoel definitions (in the past 12 months) Chi-squared	None	Sex was not associated with workplace bullying. Younger workers (18–30 years old) were more likely to be bullied (prevalence = 51%) than the others (31–40 yo = 40%; 41–50 yo = 34%; >50 yo = 35%). Bullying was more frequent among full-time workers (full-time, prevalence = 47%; part-time = 30%). Unqualified residential care staff (48%) and nurses (44%) presented higher prevalence of exposure to bullying, compared to doctors (31%), ancillary staff (27%), administrative staff (37%), therapists (37%) and psychologists (36%).				12
51	Cole [30] 1997 United States	598 participants from 2250 eligible workers (who represented the national population of fulltime workers). Response rate = 26.6%.	Cross-sectional Question about harassment directed at the respondent while at work in the past 12 months. Logistic regression	Age, gender, work climate, work structure, job uncertainty, and professional status.	Age, gender, work climate, and job uncertainty were associated with harassment. Age (19–44), OR 2.13 (1.19–3.81) Gender (female), OR 1.81 (1.15–1.82) Low co-worker support, OR 2.04 (1.16–3.62) Low work group harmony, OR 2.51 (1.52–4.13) Layoffs, OR 1.97 (1.26–3.09) Professional status and work structure were not associated with bullying.				14
